# Disease‐associated polymorphisms within the conserved ECR1 enhancer differentially regulate the tissue‐specific activity of the cannabinoid‐1 receptor gene promoter; implications for cannabinoid pharmacogenetics

**DOI:** 10.1002/humu.23931

**Published:** 2019-11-04

**Authors:** Elizabeth A. Hay, Philip Cowie, Andrew R. McEwan, Ruth Ross, Roger G. Pertwee, Alasdair MacKenzie

**Affiliations:** ^1^ School of Medicine, Medical Sciences and Nutrition, Institute of Medical Sciences, Foresterhill University of Aberdeen Aberdeen UK; ^2^ Department of Pharmacology and Toxicology University of Toronto Toronto Ontario Canada

**Keywords:** 212‐2, AP‐1, cannabinoid pharmacogenetics, CB1 agonists, chromatin immunoprecipitation, CTCF, disease‐associated polymorphisms cannabinoid‐1 receptor, enhancer, gene regulation, promoter, tissue specific, Win55

## Abstract

Cannabinoid receptor‐1 (CB1) represents a potential drug target against conditions that include obesity and substance abuse. However, drug trials targeting CB1 (encoded by the *CNR1* gene) have been compromised by differences in patient response. Toward addressing the hypothesis that genetic changes within the regulatory regions controlling CNR1 expression contribute to these differences, we characterized the effects of disease‐associated allelic variation within a conserved regulatory sequence (ECR1) in *CNR1* intron 2 that had previously been shown to modulate cannabinoid response, alcohol intake, and anxiety‐like behavior. We used primary cell analysis of reporters carrying different allelic variants of the human ECR1 and found that human‐specific C‐allele variants of ECR1 (ECR1(C)) drove higher levels of CNR1prom activity in primary hippocampal cells than did the ancestral T‐allele and demonstrated a differential response to CB1 agonism. We further demonstrate a role for the AP‐1 transcription factor in driving higher ECR1(C) activity and evidence that the ancestral t‐allele variant of ECR1 interacted with higher affinity with the insulator binding factor CTCF. The cell‐specific approaches used in our study represent an important step in gaining a mechanistic understanding of the roles of noncoding polymorphic variation in disease and in the increasingly important field of cannabinoid pharmacogenetics.

## 1 INTRODUCTION

The cannabinoid‐1 receptor (CB1) is expressed in areas of the nervous system that include the hypothalamus and the hippocampus where CB1 plays a critical role in appetite regulation (Pomorska, do‐Rego, do‐Rego, Zubrzycka, & Janecka, 2016) and neuroprotection against stress (Akirav, 2011). For this reason, CB1 has been explored as a target for drugs to treat diseases, including obesity and depression (Huang, Chen, & Zhang, 2016; Pomorska et al., 2016). However, problems with potential side effects to drugs developed to target CB1 have delayed the development of cannabinoids as potential drug therapies. For example, the synthetic CB1 antagonist rimonabant, marketed as an appetite suppressor, was withdrawn because 26% of patients reported depression, anxiety, and feeling of suicidality (Lazary, Juhasz, Hunyady, & Bagdy, 2011). The role of genetics in modulating cannabinoid response is further supported by the findings of a strong genetic component to the psychotic, cognitive, and addictive side effects of CB1 agonists (Hryhorowicz, Walczak, Zakerska‐Banaszak, Słomski, & Skrzypczak‐Zielińska, 2018; Mandelbaum & de la Monte, 2017). Given the potential benefits of the pharmacological manipulation of CB1, it is essential to gain a better understanding of cannabinoid pharmacogenetics to facilitate the development and application of safe and effective cannabinoid‐based therapeutics.

Previous studies have shown that the coding regions of the *CNR1* gene, which encodes CB1, lack common nonsynonymous polymorphisms that might account for differences in cannabinoid response. Thus, efforts to understand the regulation of the *CNR1* gene, and how it might be affected by polymorphic variation, are currently underway. For example, intron 2 of the human *CNR1* gene contains a 3‐kb linkage disequilibrium block (LD block) that contains 17 polymorphisms, two of which rs2023239 and rs9450898, are associated with addictive behaviors (Ketcherside, Noble, McIntyre, & Filbey, 2017), depression (Icick et al., 2015), psychosis (Suárez‐Pinilla et al., 2015), reduced hippocampal volume in cannabis abuse (Schacht, Hutchison, & Filbey, 2012), nicotine addiction (Chen et al., 2008), obesity (Benzinou et al., 2008), and alcohol abuse (Hutchison et al., 2008; Pava et al., 2012). Intriguingly, specific haplotypes of the human *CNR1* locus, which includes the rs2023239 locus, are associated with a significant reduction in *CNR1* expression in human hippocampus (Zhang et al., 2004) and a more recent paper demonstrated that the G‐allele of rs2023239 was associated with a greater expression of *CNR1* messenger RNA (mRNA) in peripheral lymphocytes (Ketcherside et al., 2017).

**Figure 1:**
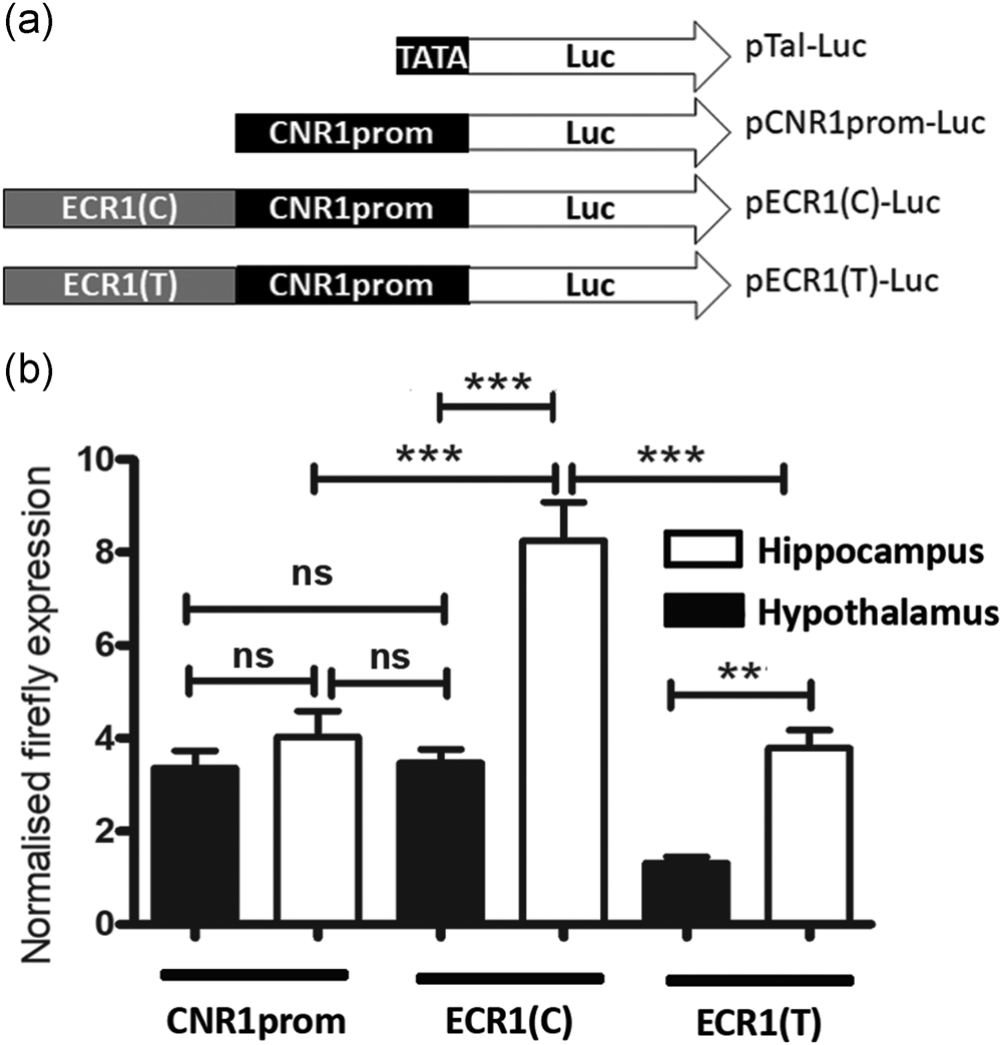
** **(a) Allelic variants of the ECR1 enhancer drive differential activity of the cannabinoid receptor promoter in different tissues. Diagrammatic representation of the reporter constructs used in the current study demonstrating the relative positions of the ECR1 element (light gray), the CNR1prom promoter sequence (black) and reporter genes (white, firefly luciferase, not to scale). (b) Dual luciferase analysis comparing the relative activity of allelic variant of the ECR1 sequence in primary hypothalamic and hippocampal cells magnetofected with pCNR1prom‐Luc (CNR1prom), pECR1(C)‐luc (ECR1(C)) or pECR1(T)‐luc (ECR1(T)) (*n* = 14, error bars = standard deviation of the mean [*SDM*], **p < .01; ***p < .005, *ns*, no significance)

Subsequent studies of the *CNR1* intron 2 LD block identified a highly conserved and active enhancer (ECR1), which contained a polymorphism (rs9444584; NC_000006.12:g.88152840C>T, NC_000006.11:g.88862559C>T) in high LD with both rs2023239 (NC_000006.12:g.88150763T>C, NC_000006.11:g.88860482T>C) and rs9450898 (NC_000006.12:g.88154344C>T, NC_000006.11:g.88864063C>T; Figure 1b; Nicoll et al., 2012). Deletion of this enhancer using CRISPR/CAS9 genome editing produced mice that expressed less hippocampal *CNR1* mRNA, that drank less alcohol, had altered levels of anxiety‐like behavior and had a blunted response to cannabinoid‐1 receptor agonism (Hay, Cowie et al., 2019; Hay, McEwan et al., 2019).

The current study examines the hypothesis that polymorphic changes in the human ECR1 enhancer affect the activity of CNR1prom in a tissue‐specific manner and also produce differential activation of CNR1prom when challenged with a CB1 agonist. We also explore the identity of the transcription factors that may interact with the polymorphic regions of ECR1 and carry out coexpression studies to determine the effects of their interactions on regulatory activity. We discuss the possibility that the findings described in this study represent an important step in our journey to understand the role of regulatory variation in cannabinoid pharmacogenetics.

## 2 MATERIALS AND METHODS

### 2.1 Bioinformatic analysis using RegSNP

Prediction of the effects of allelic variants of rs9444584 on the binding of transcription factors was predicted using the RegSNP website (http://viis.abdn.ac.uk/regsnp/Home.aspx).

### 2.2 Plasmid constructs

*pCNR1prom‐Luc*: The human genomic fragment containing the *CNR1* promoter (CNR1prom) was amplified from human placental DNA using the following primers (Zhang et al., 2004):

CNR1prom forward 5′‐GATAACCTTTTCTAACCACCCACCTAG‐3′,
CNR1prom reverse 5′‐GCGGAAAAGAAGTGGAGAAG‐3′

and cloned into the *EcoRI* and *SacI* restriction sites of the pGL4.23 luciferase reporter construct to create the pCNR1prom‐Luc.

Production of pECR1(C)CNR1promLuc and pECR1(T)CNR1promLuc firefly luciferase reporter constructs was achieved by cloning the ECR1(C) or ECR1(T) regions from the pGEM‐Teasy parent constructs using *AatII* and *SalI* sites and ligating into *AatII* and *XhoI* sites of the pCNR1prom‐Luc placing ECR1(C) or ECR1(T) upstream of the *CNR1*prom fragment to form pECR1(C)Luc and pECR1(T)Luc (Figure 1a).

### 2.3 Primary cell culture

**Figure 2:**
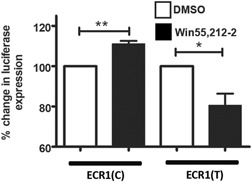
The C‐allele of the ECR1 allele permits autoregulation of the *CNR1* promoter while the T‐allele acts as a repressor. Dual luciferase analysis of primary hippocampal cells magnetofected with pECR1(C)‐luc (pECR1(C)) or pECR1(T)‐luc (pECR1(T)) constructs and treated with vehicle (white bars) or Win55,212‐2 (100 nM; black bars) for 24 hr (*n* = 6, error bars = *SDM*, *p < .05, **p < .01). Win55,212‐2 treated dual luciferase results (black bars) were normalized against the DMSO‐treated results (white bars) for clarity. *SDM*, standard deviation of the mean. *SDM*, standard deviation of the mean

One‐day‐old male and female Sprague‐Dawley neonate rats were euthanized in accordance with current UK Home Office schedule 1 guidelines. Hippocampal or hypothalamic tissues were dissected into ice cold Neurobasal‐A medium (Life Technologies). Cells were dissociated using a combination of trypsin and papain solution followed by gentle agitation from pooled tissues to reduce variability and cultured in poly‐d‐lysine coated 24‐well plates as previously described (Nicoll et al., 2012) at a density of 180,000 cells per well as assessed using a Biorad TC10 cell viability counter (Biorad). Cells were maintained at 37°C in 5% CO_2_ for up to 7 days in vitro in Neurobasal‐A medium supplemented with 2% B27, 2 mM l‐glutamine, 50 μg/ml Streptomycin, and 50 U Penicillin (Life Technologies) with the medium changed on the first day after plating and then every 3 days. Cultures were transfected 4 days after plating with luciferase plasmids (Figure 2a) plus renilla luciferase plasmid (pGL4.74) as a transfection normalization control. Neuromag transfection reagent was used as per the manufacturer's instructions (OZ Biosciences). Finally, 24 hr after transfection cultures were treated with the CB1 agonist Win55,212‐2 (Pertwee et al., 2010) for a further 24 hr.

### 2.4 Transformed cell culture and transfection

To determine whether c‐fos interacted with the ECR1 enhancer, we cotransfected different genotypes of the ECR1 enhancer driving the CNR1prom within a luciferase reporter in the presence/absence of an empty expression vector (pcDNA3) or an expression vector expressing the c‐fos protein into the SH‐SY5Y neuroblastoma cell line. SH‐SY5Y cells (94030304; ECAC, UK) were cultured in Dulbecco's modified Eagle medium (Gibco, UK) containing low glucose (5.5 mM), l‐glutamine (4 mM), and sodium pyruvate (1 mM). Medium was supplemented with 10% (v/v) heat‐inactivated fetal bovine serum (Gibco) and 1% (v/v) Penicillin–Streptomycin (Pen‐Strep; Gibco). The cells were cotransfected with luciferase reporter plasmids (Figure 2a) or a positive control (pAP1‐3; Addgene; 71258) and a renilla normalization control (pGL4.74; Promega, UK) together with pcDNA‐FLAG‐FosWT (Addgene; 8966) or empty expression vector (pcDNA3.1; Thermo Fisher Scientific; V79020) plasmid using jetPRIME as per the manufacturer's instruction (Polyplus Illkirch, France).

### 2.5 Luciferase reporter assays

All luciferase reporter assays were performed either 24 (SH‐SY5Y cells) or 48 hr (primary cells) after transfection as per the manufacturer's instructions (Promega). Luciferase expression was quantified using Dual Luciferase Reporter assay system and a GloMax 96 microplate luminometer (Promega).

Chromatin immunoprecipitation (ChIP) was performed on chromatin derived from primary hippocampal neurons transfected with either the ECR1(C) or ECR1(T) reporter constructs (1.2 µg/5 × 10^6^ cells in 10 cm plates), as described above, and incubated for 48 hr. Briefly, following cross linking with formaldehyde hippocampal chromatin was extracted and fragmented by restriction digestion (NcoI/BglII and MluI/AseI) or sonication as previously described (Carey, Peterson, & Smale, 2009) and incubated in the presence of a mouse immunoglobulin G antibody (Upstate), anti‐CTCF (Abcam) or anti‐Jun antibody (Sigma). Chromatin–antibody complexes were recovered using preblocked (1 mg/ml bovine serum albumin and 1 μg/ml salmon sperm DNA) Dynabeads Protein A (Life Technologies).

Quantitative real‐time polymerase chain reaction (PCR) for ChIP samples: quantitative PCR (qPCR) for ChIP samples was conducted using SYBR green reagents as per manufacturer's instructions (Roche) using the following primers to normalize transfection efficiencies from input DNA (LucChIP forward; CTTGCAGTTCTTCATGCCCG, LucChIP reverse; CTCACGAATACGACGGTGGG) and to assess immunoprecipitation of human ECR1T or ECR1C allelic variants following immunoprecipitation (hECR1 forward; TTAATGGCAGCTACATCCCC, hECR1 reverse; TCATGGCAGGAAAACTGCTC).

### 2.6 Data analysis

Two‐tailed unpaired parametric Student's *t* test was used to examine the difference between two groups. Where there were more than two groups, statistical significance of data sets was analyzed using a one‐way analysis of variance with Bonferroni post hoc tests. All tests were carried out using GraphPad PRISM version 5.02 (GraphPad Software, La Jolla, CA).

## 3 RESULTS

### 3.1 Allelic variants of the human ECR1 enhancer differentially regulate the activity of CNR1prom

We had previously shown that the ECR1 enhancer could stimulate the activity of a generic TATA box promoter when transfected into different primary cell types and that the T‐alelle drove stronger activity of this generic promoter (Nicoll et al., 2012). Because there is extensive evidence of enhancer‐promoter selectivity in the genome (Furlong & Levine, 2018), the current study sought to compare the interactions of these different human ECR1 variants with the previously characterized *CNR1* promoter (CNR1prom; Zhang et al., 2004). We produced a luciferase reporter construct supported by the human CNR1prom fragment (pCNR1prom‐Luc), as previously described (Zhang et al., 2004). We then cloned allelic variants of ECR1 (ECR1C and ECR1T) into the pCNR1‐Luc construct (Figure 1a) and magnetofected these constructs into rat hippocampal and hypothalamic primary cell cultures. We then carried out dual luciferase analysis of these transfected cell cultures 48 hr later. In contrast to our previous studies, where we had shown that the ancestral T‐allele drove stronger activity of the generic TATA box promoter (Nicoll et al., 2012) we observed that the T‐allele failed to enhance activity of CNR1prom in hippocampal‐derived cells and actually repressed the activity of CNR1prom in cells derived from hypothalamus. Instead, we observed strong enhancement of CNR1prom activity by the C‐allele in hippocampal cells (Figure 1b) demonstrating evidence of enhancer‐promoter specificity (Spurrell, Dickel, & Visel, 2016) in the interaction of CNR1prom and ECR1(C) in these cells.

### 3.2 Allelic variants of ECR1 differentially modulate the autoregulatory effects of CB1 agonism on the CNR1 promoter

In keeping with previous observations (Borner, Hollt, Sebald, & Kraus, 2007; Mukhopadhyay et al., 2010), we had shown that the activity of the endogenous *CNR1* gene and the activity of the CNR1prom could be upregulated through pharmacological activation of the CB1 receptor in both hippocampal‐ and hypothalamus‐derived cells suggesting the possibility of a mechanistic autoregulatory loop in the modulation of *CNR1* expression in response to its stimulation (Hay, Cowie et al., 2019; Hay, McEwan et al., 2019). Then, we asked whether the C‐ and T‐alleles of ECR1 affected the response of CNR1prom to agonist treatment. Reporter constructs containing allelic variants of the ECR1 enhancer and CNR1prom were magnetofected into hippocampal primary cell cultures which were then treated with the CB1 agonist Win55,212‐2 (100 nM) as used in previous studies (Pertwee et al., 2010). Dual luciferase analysis of these cells demonstrated that incubation of cells with Win55,212‐2 significantly increased luciferase expression in the presence of the ECR1(C) allele (Figure 2). However, we observed a significant reduction in the response of CNR1prom to Win55,212‐2 treatment in the presence of ECR1(T) suggesting that ECR1T does not support autoregulation of CNR1prom in hippocampal cells and may even repress its activity (Figure 2).

### 3.3 ECR1(C) interacts with higher affinity to the AP‐1 transcription factor than ECR1(T)

We next asked whether we could identify the putative protein–DNA interactions responsible for the ability of ECR1(C) to upregulate the activity of the CNR1 promoter. Bioinformatic analysis (RegSNP) of the human ECR1 locus predicted that the AP‐1 transcription factor (a dimer of the c‐FOS and c‐JUN proteins) would bind to ECR1(C) with high affinity but with reduced affinity to ECR1(T). The RegSNP program also suggested that the ECR1(T) allele would bind the CTCF transcription factor with higher affinity. This is intriguing as CTCF is a known marker for the function of regulatory sequences known as insulators that protect promoters against the effects of enhancer sequences (Agrawal, Heimbruch, & Rao, 2018).

**Figure 3:**
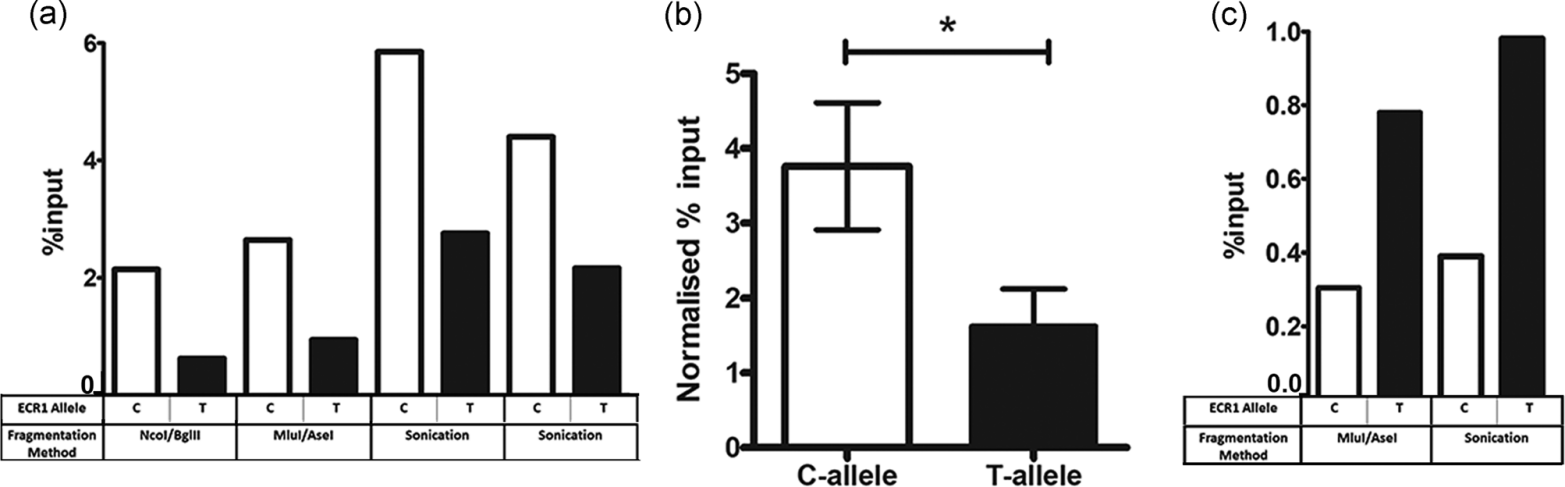
The C‐allele of ECR1 binds the c‐jun protein with higher affinity than the T‐allele. (a) Bar graph demonstrating the results of four separate ChIP assays performed on cross linked chromatin from primary hippocampal cells magnetofected with vectors containing either the ECR(C) and ECR1(T) allelic variants. Before immunoprecipitation with c‐JUN chromatin was fragmented by endonuclease digestion (NcoI/BglII or MluI/AseI) or sonication. All values have been normalized against the immunoglobulin G (IgG) immunoprecipitation signal and displayed as a percentage of the input signal. (b) Statistical analysis (two‐tailed unpaired *t* test) of all of the ChIP assays displayed in (a) demonstrating the relative levels of quantitative polymerase chain reaction signals received from DNA immunoprecipitated using the anti‐c‐JUN antibody. (c) Bar graph demonstrating the results of two separate ChIP assays carried out as described above but using an anti‐CTCF antibody. Values have been normalized against IgG controls and displayed as a proportion of input DNA (error bars = *SDM*; *p < .05). *SDM*, standard deviation of the mean

To explore these predictions, we devised a novel primary cell‐based assay that involved the magnetofection of human allelic variants of ECR1 within the ECR1(C)‐Luc or ECR1(T)‐Luc plasmids (Figure 3a) into primary rat hippocampal cell cultures. After 48 hr, chromatin was extracted and fragmented using restriction digestion or sonication (Figure 3a). qPCR of extracted chromatin using primer pairs designed to detect the luciferase gene DNA on input DNA indicated equal transfection efficiencies. The amount of luciferase DNA in the input DNA was used to normalize the qPCR signal after immunoprecipitation with antisera against the c‐JUN or CTCF proteins to ensure transfection equivalence. After recovery of specific antibody–protein–DNA complexes qPCR specific for human ECR1 was used to determine comparative levels of human ECR1 DNA immunoprecipitation. Despite using three different genome fragmentation techniques we consistently observed a higher signal from primary cells transfected with ECR1CLuc compared with those transfected with ECR1TLuc suggesting a significantly increased affinity for c‐JUN to the C‐allele (Figure 3a,b). Intriguingly, we also observed increased binding of CTCF to the T‐allele of ECR1 and were able to reproduce these observations in two separate experiments (Figure 3c).

### 3.4 c‐FOS overexpression increases the ability of ECR1(C) but not ECR1(T) to activate CNR1prom

**Figure 4:**
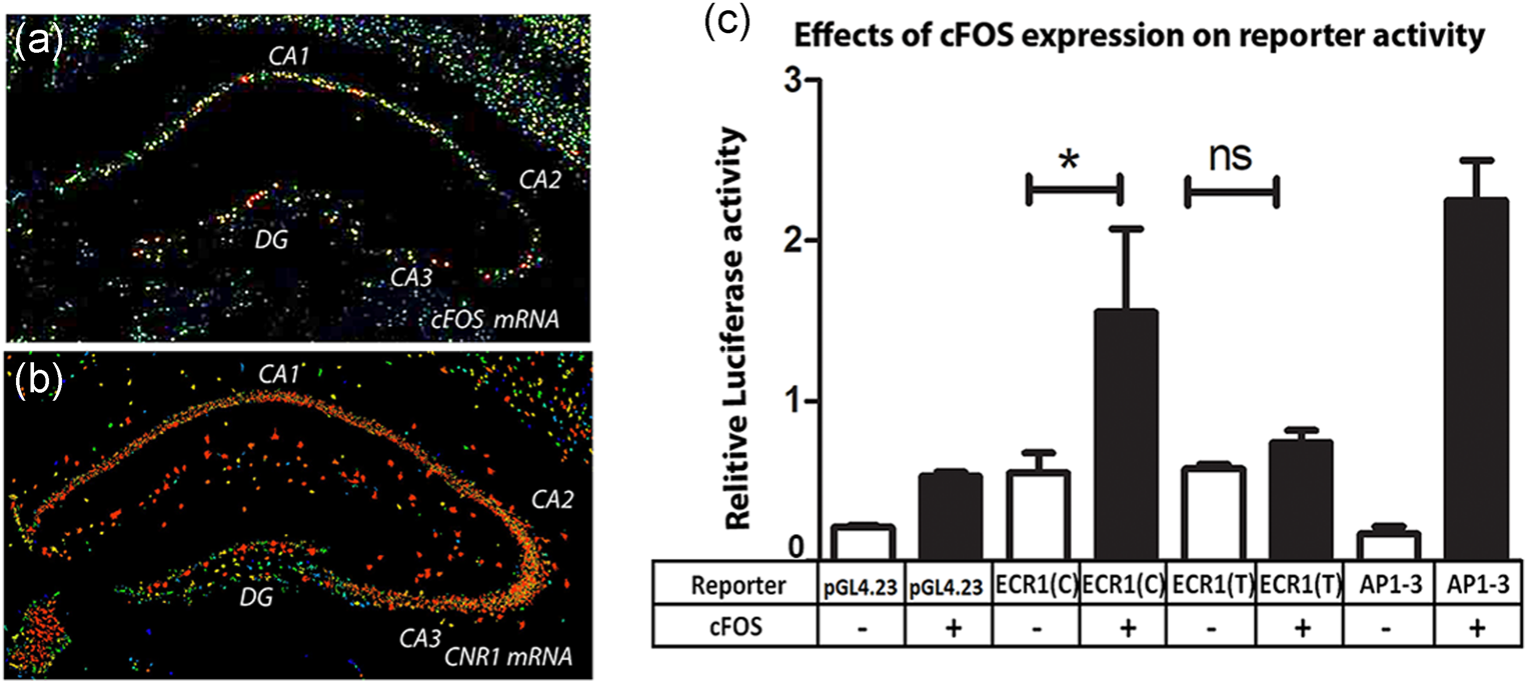
The C‐allele of ECR1 interacts more strongly with the c‐fos protein than does the T‐allele. In situ hybridization (Allen brain atlas) of a coronal section of mouse midbrain comparing localization of (a) c‐FOS and (b) *CNR1* messenger RNA (mRNA) within different regions of the hippocampus (CA1, CA2, CA3, and dentate gyrus [DG]). (c) Results of a cotransfection study in SH‐SY5Y cells where different reporter constructs (pCNR1prom‐Luc (CNR1prom), pECR1(C)‐luc (ECR1(C)) or pECR1(T)‐luc (ECR1(T)), pAP1‐3 (AP1‐3)), cotransfected with either the empty expression vector (pcDNA3.1) or an expression vector expressing the c‐FOS protein. AP1‐3; pAP1‐3‐positive control plasmids responsive to AP‐1 expression. All transfections were normalized against renilla luciferase (pGL4.74) and possible trans effects were negated by normalizing against cells transfected with empty vector (*n* = 3, error bars = *SDM, ns*, not significant, *p < .05). *SDM*, standard deviation of the mean

In addition to being a component of the AP‐1 transcription factor c‐FOS is coexpressed with *CNR1* in the hippocampus (Figure 4a) and it is known that upregulation of CB1 activity also increases activation of c‐FOS (Marie‐Claire et al., 2003; Porcella, Gessa, & Pani, 1998). To explore the possible functional relationship of c‐FOS with different allelic variants of ECR1 we carried out a cotransfection analysis in a human neuroblastoma cell line (SH‐SY5Y cells) with luciferase reporters containing the C‐allele and T‐allele of ECR1 in combination with an expression vector expressing the c‐FOS protein (pcDNA‐FosWT). We observed that coexpression of c‐FOS with a luciferase reporter containing a promoter known to respond strongly to AP‐1 (pAP1‐3) was highly upregulated (Figure 4b). We also observed a significant increase in luciferase expression in cells cotransfected with ECR1CLuc and pcDNA‐c‐FOS indicating that expression of c‐FOS drives ECR1(C) to activate CNR1prom. However, consistent with our previous primary cell transfection and ChIP data we found no significant upregulation in activity of ECR1(T) in the presence of c‐FOS expression (Figure 4b).

## 4 DISCUSSION

Although targeting the CB1 receptor has clear therapeutic potential the beneficial effects of cannabinoid drug treatments are not universal (Hryhorowicz, Walczak, Zakerska‐Banaszak, Słomski, & Skrzypczak‐Zielińska, 2017). Thus, understanding the pharmacogenetics of the *CNR1* locus within the human population is essential to the therapeutic development of the cannabinoids and being able to select which members of the population would benefit most. Because there is evidence that the majority of functional genomic variations occurs outside of coding regions (Boyle, Li, & Pritchard, 2017) and the *CNR1* coding region does not contain polymorphisms that occur in >1% of the population, the current study sought to better understand the noncoding genetic influences controlling the tissue‐specific expression of the *CNR1* gene as possible contributory factors in the pharmacogenomics of cannabinoid response.

Using a combination of linkage disequilibrium of disease‐associated SNPs and comparative genomics we identified a polymorphic enhancer sequence that we called ECR1 within intron 2 of the CNR1 locus (Nicoll et al., 2012). Subsequent analysis showed that this sequence had differential enhancer activity when cloned into a reporter construct with a short generic TATA box promoter (Nicoll et al., 2012). Disrupting the ECR1 enhancer element using CRISPR genome editing in mice demonstrated that ECR1 is important for maintaining normal levels of CNR1 expression within the hippocampus (Hay, Cowie et al., 2019; Hay, McEwan et al., 2019). This experiment also permitted in vivo behavioral studies to demonstrate the effects of ECR1 disruption on core body temperature, following CB1 activation, and ethanol intake (Hay, Cowie et al., 2019; Hay, McEwan et al., 2019). This last observation is interesting as human ECR1 contains the rs9444584 polymorphism; part of a haplotype block that has been implicated in reduced CNR1 expression (Zhang et al., 2004) and increasing susceptibility to alcohol abuse (Hutchison et al., 2008; Pava et al., 2012). Thus, in addition to its possible role in stratification of drug response, further functional analysis of this polymorphism in vivo and in the clinic may reveal important insights into the causes of alcohol abuse.

Intriguingly, our analysis of the interaction of different alleles of ECR1 with the endogenous *CNR1* promoter contrasted strongly with that previously published whereby the T‐allele of ECR1 drove stronger activity of a generic TATA box promoter (Nicoll et al., 2012). Thus, in the presence of the C‐allele of ECR1, the endogenous *CNR1* promoter was much more active that in the presence of the T‐allele in direct contrast to their interaction with the small generic TATA box promoter previously used. Indeed, to the best of our knowledge, this is the first time that such a significant difference has been observed in the effects of two different allelic variants of an enhancer on the activity of two different promoters. Moreover, the interaction of the C‐allele with the endogenous promoter is consistent with the observation that, in human hippocampus, the haplotype associated with strongest expression of *CNR1* was that containing the C‐haplotype (Zhang et al., 2004). Although the specific mechanisms involved can only be speculated on, we believe that the data derived from our analysis of the interaction of the two allelic variants of ECR1 with the *CNR1* promoter is more representative of the endogenous system so represents a more credible paradigm. This comparison also suggests that caution should be exercised by those attempting to reach conclusions pertaining to the effects of polymorphic variation in enhancers based on their interaction with foreign promoters.

From the perspective of tissue‐specific regulation our observations suggest that ECR1 may play a more substantive role in supporting expression of *CNR1* in the hippocampus than the hypothalamus implying that SNPs within ECR1 would have a greater effect in the hippocampus. This observation is consistent with previous observations in humans where a specific haplotype involving SNPs in high LD with rs9444584 significantly reduced the expression of *CNR1* in the hippocampus (Zhang et al., 2004). Considering the known neuroprotective role of CB1 activation against the effects of stress in the hippocampus (Scarante et al., 2017) it is possible that the C‐allele may play a role in protecting the hippocampus against the depressive and anxiety inducing effects of stress in humans. In this context, and from the perspective of adaptive evolution, it is interesting that the C‐allele has undergone positive selection in European and Asian populations where its frequency exceeds 80% compared with the ancestral T‐allele. Although bottleneck effects might account for this difference, we cannot rule out the possibility that selection for an allele which drives higher levels of CB1 receptor in the hippocampus may have been beneficial to early human populations moving into challenging new habitats by resisting the anxiety and depressive effects of stress.

These observed differences in the activity of the C‐ and T‐alleles of ECR1 in the hippocampus may also influence the observed effects of CB1‐targeted therapeutics such as the antiobesity drug rimonabant in humans. Rimonabant's main mode of action is through antagonism/inverse agonism of the CB1 receptor in the hypothalamus, which leads to a reduction in appetite. Rimonabant was withdrawn from the market as it was linked to an increase in depressed and suicidal feelings in 26% of patients (Lazary et al., 2011). If we consider what is known about the neuroprotective role of CB1 against stress in the hippocampus, the increase in anxiety and depression in these individuals is not surprising as the appetite reducing antagonism of CB1 in the hypothalamus would be accompanied by antagonism of CB1 in the hippocampus. Indeed, reports of increased anxiety (O'Brien et al., 2013) and depression‐like (Beyer et al., 2010) behaviors have been reported in rodents, who harbor the ancestral T‐allele of ECR1, following chronic administration of rimonabant. What is probably more noteworthy is the observation that 74% of patients did not experience anxiety/depression‐like symptoms and responded positively to rimonabant. Based on our observations, we propose that the human‐specific C‐allele of ECR1 may induce higher levels of CB1 expression in the hippocampus in humans, thus protecting individuals from the anxiolytic and depression forming effects of stress following treatment with rimonabant (Akirav, 2011).

Our analysis goes on to identify a possible molecular mechanism that may explain differences in the activity of the C‐ and T‐alleles of ECR1 based on variable affinity to the c‐JUN and c‐FOS components of the AP‐1 transcription factor which is known to be expressed in the hippocampus. Using a unique experiment based on ChIP analyses of magnetofected primary hippocampal cells we demonstrated increased affinity for c‐JUN to the C‐allele of ECR1(C). To verify this observation, we also showed that expression of c‐FOS; one of the proteins that forms the AP‐1 complex, activates the ECR1(C) enhancer but not ECR1(T) in cotransfected cells. This observation was interesting as several studies have demonstrate that stimulation of CB1 induces binding of AP‐1 to DNA and expression of c‐FOS (Marie‐Claire et al., 2003; Porcella et al., 1998). This was particularly evident in the hippocampus where CB1 and c‐FOS are coexpressed in the CA1, CA3, and dentate gyrus (Derkinderen et al., 2003).

Our observations also touch on the evolution of the C‐allele which is only found in humans. We were able to predict binding of the CTCF transcription factor; a known marker of insulator function, with higher affinity to the T‐allele, a prediction that we also support using ChIP assays. These observations raise an interesting possibility that the ancestral T‐allele of ECR1 could act as a weak enhancer with insulator properties in the hippocampus of most vertebrates. However, the change to the C‐allele in humans, that increased binding affinity of AP‐1 and increased the enhancer like properties of ECR1 in specific tissues, may have proved advantageous to specific populations faced with new and stressful circumstances thus expanding the frequency of the C‐allele in these populations.

Taken together, the evidence discussed above suggests a role for allelic variation within ECR1 in modulating the activity of CNR1prom and thus expression of *CNR1* and a mechanism is proposed based on the evidence. Although much remains to be done to conclusively establish a role for these observations in alcohol abuse and drug effects in humans, our unique primary cell‐based ChIP transfection studies and reporter‐expression vector coexpression studies lay the foundation for future studies of the contribution of these genetic and epigenetic factors in the pharmacogenetics of the cannabinoids and the possible effects on human health and disease susceptibility. We believe that the current manuscript provides not only a platform for the further study of the effects of polymorphisms on the pharmacogenomics of the cannabinoids but also an important functional tool for understanding the role of noncoding polymorphisms in health and disease.

## Data Availability

The data that support the findings of this study are available from the corresponding author upon reasonable request.
